# Local Functional MR Change Pattern and Its Association With Cognitive Function in Objectively-Defined Subtle Cognitive Decline

**DOI:** 10.3389/fnagi.2021.684918

**Published:** 2021-06-11

**Authors:** Liang Cui, Zhen Zhang, Chun-Yi Zac Lo, Qihao Guo

**Affiliations:** ^1^Department of Gerontology, Shanghai Jiao Tong University Affiliated Sixth People’s Hospital, Shanghai, China; ^2^Institute of Science and Technology for Brain Inspired Intelligence, Fudan University, Shanghai, China

**Keywords:** objectively-defined subtle cognitive decline, mild cognitive impairment, function activity, resting-state functional MRI, cognitive function

## Abstract

**Introduction**: To identify individuals with preclinical cognitive impairment, researchers proposed the concept of objectively-defined subtle cognitive decline (Obj-SCD). However, it is not clear whether Obj-SCD has characteristic brain function changes. In this study, we aimed at exploring the changing pattern of brain function activity in Obj-SCD individuals and the similarities and differences with mild cognitive impairments (MCI).

**Method**: 37 healthy control individuals, 25 Obj-SCD individuals (with the impairment in memory and language domain), and 28 aMCI individuals were included. Resting-state fMRI and neuropsychological tests were performed. fALFF was used to reflect the local functional activity and compared between groups. Finally, we analyzed the correlation between the fALFF values of significantly changed regions and neuropsychological performance.

**Results**: We found similar functional activity enhancements in some local brain regions in the Obj-SCD and aMCI groups, including the left orbital part of the inferior frontal gyrus and the left median cingulate and paracingulate gyri. However, some changes in local functional activities of the Obj-SCD group showed different patterns from the aMCI group. Compared with healthy control (HC), the Obj-SCD group showed increased local functional activity in the right middle occipital gyrus, decreased local functional activity in the left precuneus and the left inferior temporal gyrus. In the Obj-SCD group, in normal band, the fALFF value of the right middle occipital gyrus was significantly negatively correlated with Mini-Mental State Examination (MMSE) score (*r* = −0.450, *p* = 0.024) and Animal Verbal Fluency Test (AFT) score (*r* = −0.402, *p* = 0.046); the left inferior temporal gyrus was significantly positively correlated with MMSE score (*r* = 0.588, *p* = 0.002). In slow-4 band, the fALFF value of the left precuneus was significantly positively correlated with MMSE score (*r* = 0.468, *p* = 0.018) and AFT score (*r* = 0.600, *p* = 0.002). In the aMCI group, the fALFF value of the left orbital part of the inferior frontal gyrus was significantly positively correlated with Auditory Verbal Learning Test (AVLT) long delay cued recall score (*r* = 0.506, *p* = 0.006).

**Conclusion**: The Obj-SCD group showed a unique changing pattern; the functional changes of different brain regions have a close but different correlation with cognitive impairment, indicating that there may be a complex pathological basis inside. This suggests that Obj-SCD may be a separate stage of cognitive decline before aMCI and is helpful to the study of preclinical cognitive decline.

## Introduction

In the study of preclinical Alzheimer’s disease (AD), some researchers have proposed that there is another kind of cognitive impairment in addition to SCD (subjective cognitive dysfunction), namely Objectively-defined subtle cognitive decline (Obj-SCD). In individuals with Obj-SCD, only subtle cognitive decline can be objectively detected; they have a roughly normal cognitive function and have no memory decline complaints. Obj-SCD is defined using six neuropsychological test indicators, including two memory function indicators, two language function indicators, and two attention/execution indicators. Memory function indicators include: total score of Auditory Verbal Learning Test (AVLT) 20-min free delayed recall and total score of AVLT recognition language function indicators includes: Animal Fluency (total score) and 30-item Boston Naming Test (BNT; total score) attention/execution indicators includes: Trail Making Test (TMT) A and Trail Making Test B (time to completion). An individual is defined as Obj-SCD if they meet the following conditions: (1) Does not meet the standards of Mild cognitive impairments (MCI); and (2) In two of the three different cognitive domains (memory, language, attention/execution), only one indicator is impaired (>1 SD below demographically adjusted mean; Thomas et al., 2018, 2020).

Currently, the studies on Obj-SCD are mainly focused on preclinical AD. AD is considered a continuous process that can be diagnosed before clinical symptoms appear (Dubois et al., 2016). The National Institute on Aging and Alzheimer’s Association (NIA-AA) divided AD’s cognitive decline procession into six stages; Obj-SCD locates in the middle state between normal cognitive function and mild cognitive impairment (MCI; Jack et al., 2018). A 10-year longitudinal study based on the AD Neuroimaging Initiative showed that individuals with Obj-SCD progressed to MCI 2.5–3.4 times faster than the normal group (Thomas et al., 2018). An arterial spin labeling MRI study reported that compared with the normal cognitive function group, the cerebral blood flow of the Obj-SCD participants increased in the hippocampus and inferior parietal; compared with the MCI group, the cerebral blood flow increased in the hippocampus, inferior parietal, and inferior temporal (Thomas et al., 2021). A longitudinal study for 4 years found that Obj-SCD amyloid protein accumulated faster and showed faster thinning of the internal olfactory cortex than the normal group (Thomas et al., 2020). However, some researchers have used Obj-SCD to describe the early cognitive decline stage due to other causes, such as Parkinson’s dementia (PD). A study of PD patients revealed that participants in the Obj-SCD stage are more likely to progress to MCI due to PD or dementia due to Parkinson’s disease within 5 years (Jones et al., 2021).

Amnestic MCI (aMCI) is a subtype of MCI (Petersen, 2011). AMCI can remain stable or progress to AD; it may also develop into other forms of dementia (Petersen et al., 2001; Caminiti et al., 2018; Cerami et al., 2018; Curiel Cid et al., 2020). Individuals with aMCI have objective memory disorders, subjective memory complaints, and slightly impaired daily activities; all of the above can be detected by neuropsychological tests.

AMCI individuals have two impaired indicators in the memory function domain, therefore, there are similarities and differences between memory function impaired Obj-SCD (single memory function indicator and single another cognitive domain indicator) and single-domain aMCI (only two indicators of memory function are impaired). In terms of neuropsychological performance, cognitive impairment of the single-domain aMCI exists only in the memory domain and is severer than that of Obj-SCD.

There are many core biomarkers used in the diagnosis of cognitive impairment. The guidelines on AD proposed by NIA-AA define the biomarkers for early diagnosis of AD as A/T/N regimens, including Aβ42, total Tau, phosphorylated Tau in cerebrospinal fluid, and the detected value of Aβ42 and Tau by PET CT (Scheltens et al., 2016; Jack et al., 2018). The abnormal accumulation of α-synuclein aggregates can be used as a biomarker of some non-AD pathological dementia, such as PD, dementia with Lewy bodies, and multiple system atrophy (Manne et al., 2019). However, some of these biomarkers are too expensive, and some can only be detected through invasive tests; therefore, they are challenging to be popularized and widely adopted.

This calls for the development of a relatively simple and non-invasive method that is needed to detect early cognitive impairment. MRI can be used to screen individuals at risk of cognitive impairment (Dubois et al., 2016). In the various research methods of MRI, Resting-state functional magnetic resonance imaging (rs-fMRI) has been widely applied in the study of cognitive decline (Pan et al., 2017; Bi et al., 2020a,b; Moguilner et al., 2020). A functional connectivity study showed that the normal cognitive individuals with amyloid positive are characterized by decreased functional connectivity between the medial temporal lobe and the anterior temporal lobe system (Berron et al., 2020). Another study on default mode network (DMN) connection found that the change of functional connection mode in AD is mainly in the SLOW-4 and SLOW-5 bands; the change is frequency-dependent (Li et al., 2017). However, functional connections can only reflect the connection between different brain regions and cannot measure spontaneous activity intensity in a particular brain region (Jia et al., 2020).

Therefore, there is a need for an indicator that reflects the characteristics of local brain activity. The human brain produces numerous oscillatory waves; low-frequency fluctuation amplitude (ALFF) can reflect brain oscillatory activity’s local characteristics (Zou et al., 2008). However, ALFF is easily disturbed by physiological noise. Compared with ALFF, fractional ALFF (fALFF) can reflect the relative contribution of low-frequency fluctuations in a specific frequency band to the whole detectable frequency range, and it is not easily affected by noise (Zuo et al., 2010).

However, few studies on Obj-SCD based on fMRI have been conducted so far. We speculate that the reason might be that Obj-SCD is in the very early stage of cognitive decline, and thus detection of minor pathological changes using conventional fMRI methods is difficult. Therefore, we want to study the change of fALFF in multiple frequency bands through frequency division. We also included patients with aMCI in order to understand the similarities and differences between the Obj-SCD and the aMCI group.

This study mainly used fALFF to explore the changing pattern of regional brain function activities of Obj-SCD individuals in different frequency bands and explore the similarities and differences of the patterns between Obj-SCD and aMCI groups. We hypothesized that if Obj-SCD is indeed a unique stage before aMCI, then the local neural activity may have changes similar to aMCI, but some changes may also be different from aMCI. Since Obj-SCD may be in the pathological stage before aMCI, this difference may be related to functional compensation. To prove this hypothesis, we also analyzed the correlation between functional activity and neuropsychological performance.

## Materials and Methods

### Participants

In this study, 90 participants from local communities were recruited, including 37 healthy control individuals, 25 Obj-SCD individuals, and 28 aMCI individuals. Recruitment was carried out through advertising from August 2018 to November 2019.

Participants in this study had to meet the following criteria: (1) Chinese speakers; (2) have no history of disease that seriously affects brain function, such as craniocerebral injury, brain tumor, cerebral hemorrhage, cerebral infarction, and other systemic diseases that affect brain function (such as vitamin B12 deficiency and syphilis); (3) can complete neuropsychological tests, have no severe hearing, and visual impairment; and (4) can complete the examination of craniocerebral MRI.

Inclusion criteria of the healthy control (HC) group: (1) Mini-Mental State Examination (MMSE) score (illiteracy > 19, 1–6 education years >22, more than six education years > 26; Katzman et al., 1988); (2) a Clinical Dementia Rating (CDR) score = 0 (Morris, 1993); (3) Hamilton Depression Rating Scale score of ≤12 (Worboys, 2013); (4) no memory complaints; and (5) no evidence of memory loss provided by the observer.

Inclusion criteria of Obj-SCD group: (1) not meet Jak/Bondi criteria for MCI (Bondi et al., 2014); and (2) have and only have one impaired indicator (>1 SD below demographically adjusted mean) in two different cognitive domains (memory, language, attention/executive; Thomas et al., 2018, 2020). Neuropsychological tests used to diagnose Obj-SCD were as follows. Two measures of language: Animal Fluency (total score) and 30-item BNT (total score), two scores from a measure of attention/executive function: Trail Making Test, Parts A and B (time to completion); two scores from a measure of memory: Auditory Verbal Learning Test (AVLT) 20-min free delayed recall and AVLT recognition. For the consistency of the cohort, we included individuals with cognitive impairment in memory and language domains.

Inclusion criteria of the aMCI group: (1) with evidence of subjective memory complaints in the past year either by themselves or from bystanders; (2) MMSE above cut-off (>24/30); (3) objective memory impairment: two indicators of AVLT (long–delay free recall and recognition of AVLT) lower than the normal average of age correction >1 SD; (4) less than one item in the Activity of Daily Living Scale (ADL) changed; and (5) according to the NIA-AA criteria, there is no evidence of dementia (Bondi et al., 2014).

### Neuropsychological Tests

All the individuals participated in the following neuropsychological tests: General cognitive function: MMSE (total score: 30; Folstein et al., 1975).

Memory function: Auditory Verbal Learning Test (AVLT; score: 12 per round, immediate recall score equals the sum of the first, second, and third recall scores, recognition score: 24; Zhao et al., 2015); Brief Visuospatial Memory Test (BVMT; score: 12 per round, immediate recall score equals the sum of the first, second and third recall scores; Pliskin et al., 2020).

Language function: Animal Verbal Fluency Test (AFT; Zhao et al., 2013a), BNT (total score: 30; Mack et al., 1992).

Executive function: Shape Trail Test (STT; Zhao et al., 2013b), Stroop Test (total score: 24; Chen et al., 2019). For cultural fairness, we use STT instead of TMT to evaluate executive function.

Spatial Function: Judgment of Line Orientation (JLO; total score: 30; Qualls et al., 2000).

Attention function: Digit Span Test (DST; forward score: 12; backward score: 10; Johansson and Berg, 1989).

### Functional Magnetic Resonance Imaging

#### MRI Data Acquisition

All Resting-state fMRI images were collected using a 3.0-Tesla scanner (SIEMENS MAGNETOM Prisma 3.0 T, Siemens, Erlangen, Germany). Before the scans, the participants were told to close their eyes, stay relaxed, do not fall asleep, and move as little as possible. The image is obtained through an echo plane imaging sequence with the following parameters: repetition time (TR)/echo time (TE), 800/37 ms, flip angle (FA), 52°, matrix size, 104 × 104, the field of view, 208 mm × 208 mm, slice number, 72 slices, slice thickness, 2 mm, voxel size, 2 mm × 2 mm × 2 mm. It took 404 s to get 488 slices through scanning.

#### Imaging Data Processing

All functional imaging data were processed by Statistica Parametric Mapping 12 (SPM12)^1^ and RESTplus^2^ toolkits. The first 30 time points were discarded to stabilize the magnetic field and make the participants adjust to the environment. Then, the following preprocessing steps were performed: realign the head motion (participants whose head movement over 3 mm or more 3° had been excluded), spatially normalized to the Montreal Neurological Institute (MNI) space and resampled to 3 mm isotropic voxels, remove linear and quadratic trends of the time-series signals, regress out the sign (including white matter, cerebrospinal fluid, global mean signal, and Friston-24 motion parameters), the smoothing was done by Full Wave at Half Maximum 6 mm.

The preprocessed data were imported into the RESTplus toolkit and the fALFF in the normal band (0.01–0.08 Hz), slow4 band (0.027–0.073 Hz), and slow5 band (0.01–0.027 Hz) were calculated. For standardization, each voxel’s fALFF values were divided by the global average fALFF values of all voxels in the whole brain to obtain each participant’s mfALFF map.

### Statistical Analysis

Demographic and neuropsychological test scores were analyzed using SPSS (IBM SPSS Statistics, Version 26.0. IBM Corp., Armonk, NY, USA). The normality test of the data was performed using the Shapiro—Wilke normality test. Mean and the standard deviation was used to represent normally distributed data. Median (quartile range) is used to represent non-normally distributed data. Differences in age, education years, sex, hypertension, diabetes, and hyperlipidemia were analyzed using the Pearson chi-square test. ANOVA test was used to analyze neuropsychological test scores that fit the normal distribution between the three groups. Nonparametric tests (the Kruskal-Wallis H test) were used to analyze neuropsychological test scores that did not fit the normal distribution between the three groups. Bonferroni’s correction was used for multiple comparisons in posthoc analysis. Spearman rank correlation analysis was used to analyze the correlation between mfALLF and neuropsychological test scores.

RESTplus toolkit was used in the statistical analysis of image data. To accurately display the similarities and differences between the three groups, we conducted ANOVA analysis on the mFalff diagrams of the three groups, the threshold was set to 0.05, and a binary mask was obtained to limit the range of comparison between the groups. Multiple comparison corrections were performed using the false discovery rate (FDR) for the comparison between groups. Since multiple comparisons were involved, the FDR correction threshold was set to 0.017 to reduce the false-positive rate.

The mfALFF values of the significant clusters were extracted and correlated with neuropsychological tests by Spearman rank analysis.

## Results

### Demographic Data and Neuropsychological Performances

There were no statistical differences in age, sex, and education years among the three groups. There were no statistical differences in hypertension (24.3% in HC group, 40.0% in SCD group, 32.1% in aMCI group), hypercholesterolemia (8.1% in HC group, 16.0% in SCD group, 10.7% in aMCI group), and diabetes (13.5% in HC group, 20.0% in SCD group, 14.3% in aMCI group) among the three groups (Table 1).

**Table 1:** Demographic data and neuropsychological tests between groups.

	HC (*n* = 37)	OBJ-SCD (*n* = 25)	aMCI (*n* = 28)	Test statistic
Age (year)^(1)^	63.86 ± 8.250	64.12 ± 6.978	65.71 ± 6.895	0.531
Sex (Male/Female)^(2)^	15/22	11/14	13/15	0.231
Edu years (year)^(1)^	12.11 ± 3.422	10.84 ± 2.511	12.19 ± 3.163	1.575
Hypertension^(2)^	24.3%	40.0%	32.1%	1.731
Hypercholesterolemia^(2)^	8.1%	16.0%	10.7%	0.947
Diabetes^(2)^	13.5%	20.0%	14.3%	0.528
**General cognitive function**
MMSE^(3)^	29 (28, 30)	27 (26, 29)*	27 (26, 28)*	16.268
**Memory function**
AVLT immediate recall^(3)^	18 (15.5, 20.5)	13 (8, 16)**	12 (11, 14)**	34.144
AVLT short delay free recall^(3)^	6 (5, 8)0	4 (2, 6)**	3 (2, 2.75)**	39.854
AVLT long delay free recall^(3)^	6 (4.5, 7.5)	3 (2, 4)**	2 (1, 3)**	45.121
AVLT long delay cued recall^(3)^	6 (5, 7.5).	3 (2, 4)**	2 (2, 3)**	47.269
AVLT recognition^(3)^	22 (21, 23)	21 (19, 23)	18 (16, 18.75)**	52.679
BVMT immediate recall^(3)^	22 (16.5, 26)	14 (11, 25)	18 (14.25, 20.75)	7.646
BVMT 4th recall^(3)^	10 (7.5, 11.5)	8 (6, 10)	8 (5.25, 10)	6.806
BVMT 5th recall^(3)^	10 (8, 11.5)*	8 (6, 11)	8 (5.25, 10)	7.100
BVMT 6th recall^(3)^	5 (5, 6)0	5 (4, 6).	4 (4, 4) ^**,†^	17.112
BVMT recognition^(3)^	12 (12, 12)	12 (10, 12)*	12 (10, 12)**	15.652
**Language function**
AFT^(3)^	19 (18.5, 21.0)	15 (13, 17)**	16 (14, 17.75)**	27.372
BNT^(3)^	26 (24, 27)	23 (20, 24)**	23 (21.25, 26)*	16.866
**Executive function**				
STT-A total time (second)^(3)^	40 (34, 51)	48 (41.5, 53)	52.5 (39.25, 61.25)*	9.335
STT-B total time (second)^(3)^	113 (89, 137)	124 (107.5, 153)	127.5 (109.25, 171.00)	3.570
Stroop test A^(3)^	24 (24, 24)	24 (24, 24)	24 (24, 24)	2.107
Stroop test B^(3)^	24 (23, 24)	24 (23, 24)	23 (20, 24)*	10.893
**Spatial Function**				
JLO^(1)^	21.2 ± 5.04	19.79 ± 4.872	21.4 ± 4.32	1.526
**Attention function**				
DST sequence^(3)^	8 (7.5, 8.5)	8 (7, 8)	7 (5, 8)*	8.213
DST reverse^(3)^	5 (4, 6)0	5 (4, 6)	4.5 (4, 5)	3.519

^(1)^ANOVA test; ^(2)^Chi-square test; ^(3)^Kruskal–Wallis H-test; *compared with HC group, *p* < 0.05; **compared with HC group, *p* < 0.001; ^†^compared with Obj-SCD group, *p* < 0.05. MMSE, mini-mental state examination; AVLT, auditory verbal learning test; BVMT, brief visuospatial memory test; AFT, animal verbal fluency test; BNT, boston naming test; STT, shape trail test; JLO, judgment of line orientation; DST, digit span test. Bonferroni correction for post-hoc analysis.

All the Obj-SCD individuals included in the study showed impairment of a single neuropsychological test indicator of memory function and language function. Compared with HC group, Obj-SCD group had worse performance in MMSE [27 (26, 29) vs. 29 (28, 30)], AVLT immediate [13 (8, 16) vs. 18 (15.5, 20.5)], AVLT short delay free recall [4 (2, 6) vs. 6 (5, 8)], AVLT long delay free recall [3 (2, 4) vs. 6 (5, 8)], AVLT long delay cued recall [3 (2, 4) vs. 6 (5, 7.5)], BVMT recognition [12 (10, 12) vs. 12 (12, 12)], AFT [15 (13, 17) vs. 19 (18.5, 21)], and BNT [23 (20, 24) vs. 26 (24, 27); Table 1].

Compared with HC group, aMCI group had worse performance in MMSE [27 (26, 28) vs. 29 (28, 30)], AVLT immediate [12 (11, 14) vs. 18 (15.5, 20.5)], AVLT short delay free recall [3 (2, 2.75) vs. 6 (5, 8)], AVLT long delay free recall [2 (1, 3) vs. 6 (5, 8)], AVLT long delay cued recall [2 (2, 3) vs. 6 (5, 7.5)], AVLT recognition [18 (14.25, 20.75)], BVMT 6th recall [4 (4, 4) vs. 5 (5, 6)], BVMT recognition [12 (10, 12) vs. 12 (12, 12)], AFT [16 (14, 17.75) vs. 19 (18.5, 21)], BNT [23 (21.25, 26) vs. 26 (24, 27)], STT-A total time [52.5 (39.25, 61.25) vs. 40 (34, 51)], Stroop test B [23 (20, 24) vs. 24 (23, 24)], and DST sequence [7 (5, 8) vs. 8 (7.5, 8.5); Table 1].

Compared with the Obj-SCD group, the aMCI group had worse performance in BVMT 6^th^ recall [4 (4, 4) vs. 5 (4, 6); Table 1].

### Regional Functional Activity

#### The Obj-SCD Group Compared with the HC Group

**Figure 1:**
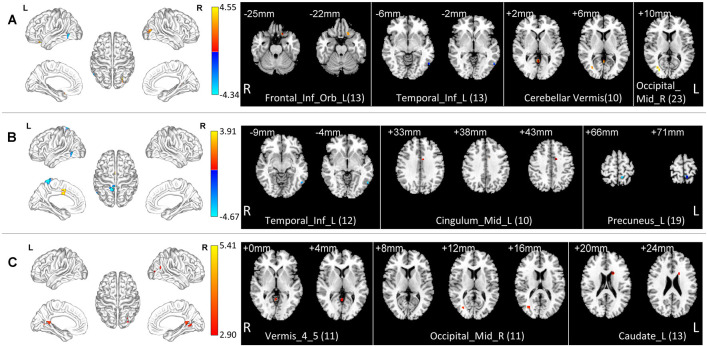
Local functional change pattern of Obj-SCD group. The Obj-SCD group compared with the HC group. **(A)** In the normal band, fALFF increased in the left orbital part of inferior frontal gyrus, the right middle occipital gyrus, cerebellar vermis; decreased fALFF in the left inferior temporal gyrus (Two-tailed *t*-test; FDR *p* < 0.017, *k* > 10 voxels). **(B)** In the slow-4 band, fALFF increased in the left median cingulate and paracingulate gyri; decreased in the left inferior temporal gyrus and the left precuneus (Two-tailed *t*-test; FDR *p* < 0.017, *k* > 10 voxels). **(C)** In the slow-5 band, fALFF increased in the right middle occipital gyrus, caudate nucleus, and cerebellar vermis (Two-tailed *t*-test; FDR *p* < 0.017, *k* > 10 voxels). The numbers in brackets are voxels of significant clusters. Labels of significant clusters are defined with the AAL (Anatomical Automatic Labeling) atlas. Abbreviations: Obj-SCD, objectively-defined subtle cognitive decline; fALFF, low-frequency fluctuation amplitude (ALFF); HC, healthy control; FDR, false discovery rate.

In the normal band, compared with the HC group, the Obj-SCD group showed increased fALFF in the left orbital part of inferior frontal gyrus, the right middle occipital gyrus, cerebellar vermis; decreased fALFF in the left inferior temporal gyrus (Two-tailed *t*-test; FDR *p* < 0.017, *k* > 10 voxels; Table 2, Figure 1).

**Table 2:** Regions with changed functional activity (fALLF) between groups.

	Normal band		Slow-4 band		Slow-5 band	
	CLUSTER (AAL)	Volume (voxels)	CLUSTER (AAL)	Volume (voxels)	CLUSTER (AAL)	Volume (voxels)
OBJ-SCD vs. HC	**Cluster 1**	total: 13	**Cluster 1**	total: 12	**Cluster 1**	total: 11
	Peak (MNI): −18 21 −24 Peak t: 4.4407		Peak (MNI): −57 −63 −12 Peak t: −4.1945		Peak (MNI): 0 −54 3 Peak t: 4.6911	
	Frontal_Inf_Orb_L	10	Temporal_Inf_L	6	Vermis_4_5	11
	**Cluster 2**	total: 13	**Cluster 2**	total: 10	**Cluster 2**	total: 11
	Peak (MNI): −57 −66 −6 Peak t: −4.4341		Peak (MNI): −12 0 42 Peak t: 3.9132		Peak (MNI): 30 −72 15 Peak t: 4.0551	
	Temporal_Inf_L	4	Cingulum_Mid_L	6	White Matter	11
					Occipital_Mid_R	4
	**Cluster 3**	total: 10	**Cluster 3**	total: 19	**Cluster 3**	total: 13
	Peak (MNI): 0 −57 3 Peak t: 4.5502		Peak (MNI): −9 −51 75 Peak t: −4.6652		Peak (MNI): −18 12 21 Peak t: 5.4060	
	Vermis_4_5	8	Precuneus_L	18	Caudate_L	4
	**Cluster 4**	total: 23
	Peak (MNI): 30 −72 15 Peak t: 3.9545
	White Matter	23
	Occipital_Mid_R	10
	**Cluster 5**	total: 11
	Peak (MNI): 30 −69 24 Peak t: 4.5089
	Occipital_Mid_R	8				
∣rule
aMCI vs. HC	**Cluster 1**	total: 10			**Cluster 1**	total: 13
	Peak (MNI): −18 21 −21 Peak t: 4.1979				Peak (MNI): 6 12 33 Peak t: 4.8347	
	Frontal_Inf_Orb_L	8			Cingulum_Mid_R	10
	**Cluster 2**	total: 14
	Peak (MNI): 6 9 33 Peak t: 4.4992
	Cingulum_MID_R	13
	**Cluster 3**	total: 13
	Peak (mni):-12 0 36 Peak t: 4.7156
	Cingulum_MID_L	7
∣rule
OBJ-SCD vs. aMCI	**Cluster 1**	total: 14
	Peak (MNI): 30 −78 12 Peak t: 3.5639
	Occipital_MID_R	8				

*AAL, anatomical automatic labeling; MNI, montreal neurological institute; fractional low-frequency fluctuation amplitude (ALFF)*.

In the slow-4 band, compared with the HC group, the Obj-SCD group showed increased fALFF in the left median cingulate and paracingulate gyri; decreased fALFF in the left inferior temporal gyrus, and the left precuneus (Two-tailed *t*-test; FDR *p* < 0.017, *k* > 10 voxels; Table 2, Figure 1).

In the slow-5 band, compared with the HC group, the Obj-SCD group showed increased fALFF in the right middle occipital gyrus, caudate nucleus, and cerebellar vermis (Two-tailed *t*-test; FDR *p* < 0.017, *k* > 10 voxels; Table 2, Figure 1).

#### The aMCI Group Compared with the HC Group

**Figure 2:**
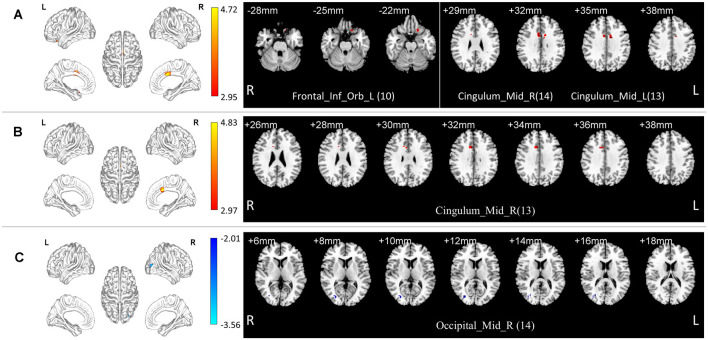
Local functional change pattern of aMCI group. **(A)** In the normal band, compared with the HC group, the aMCI group showed increased fALFF in the left orbital part of the inferior frontal gyrus, bilateral median cingulate, and paracingulate gyri (Two-tailed *t*-test; FDR *p* < 0.001, *k* > 10 voxels). **(B)** In the slow-5 band, compared with the HC group, the aMCI group showed increased fALFF in the right median cingulate and paracingulate gyri (Two-tailed *t*-test; FDR *p* < 0.001, *k* > 10 voxels). **(C)** In the normal band, compared with the Obj-SCD group, the aMCI group showed decreased fALFF in the right middle occipital gyrus (Two-tailed *t*-test; FDR *p* < 0.017, *k* > 10 voxels). The numbers in brackets are voxels of significant clusters. Labels of significant clusters are defined with the AAL (Anatomical Automatic Labeling) atlas. Abbreviation: aMCI, amnestic MCI.

In the normal band, compared with the HC group, the aMCI group showed increased fALFF in the left orbital part of the inferior frontal gyrus, bilateral median cingulate, and paracingulate gyri (Two-tailed *t*-test; FDR *p* < 0.001, *k* > 10 voxels; Table 2, Figure 2).

In the slow-5 band, compared with the HC group, the aMCI group showed increased fALFF in the right median cingulate and paracingulate gyri (Two-tailed *t*-test; FDR *p* < 0.001, *k* > 10 voxels; Table 2, Figure 2).

#### The Obj-SCD Group Compared with the aMCI Group

In the normal band, compared with the aMCI group, the Obj-SCD group showed increased fALFF in the right middle occipital gyrus (Two-tailed *t*-test; FDR *p* < 0.017, *k* > 10 voxels; Table 2, Figure 2).

### Correlation Analysis

**Figure 3:**
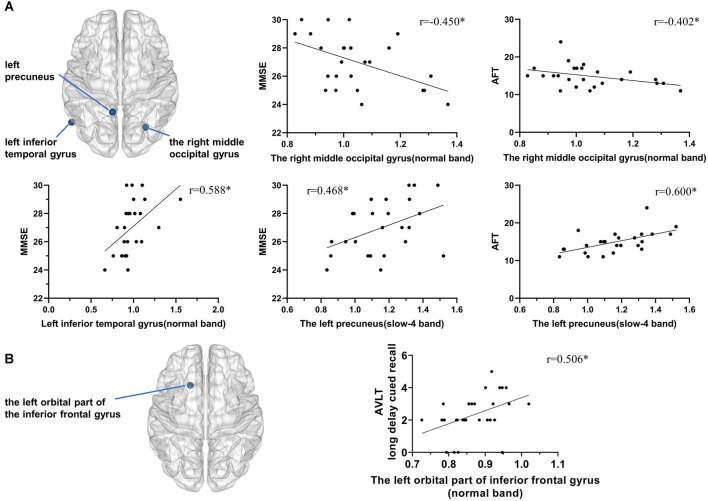
Correlation between local functional activity and neuropsychological performance. **(A)** In the Obj-SCD group, in the normal band, the fALFF value of the right middle occipital gyrus was significantly negatively correlated with the MMSE score and AFT score; the left inferior temporal gyrus was significantly positively correlated with the MMSE score. In the slow-4 band, the fALFF value of the left precuneus was significantly positively correlated with the MMSE and AFT scores. **(B)** In the aMCI group, the fALFF value of the left orbital part of the inferior frontal gyrus was significantly positively correlated with AVLT 6th recall score. *p < 0.05. Abbreviations: AFT, Animal Verbal Fluency Test; AVLT, Auditory Verbal Learning Test; MMSE, Mini-Mental State Examination.

In the Obj-SCD group, in normal band, the fALFF value of the right middle occipital gyrus was significantly negatively correlated with MMSE score (*r* = −0.450, *p* = 0.024) and AFT score (*r* = −0.402, *p* = 0.046; Figure 3); the left inferior temporal gyrus was significantly positively correlated with MMSE score (*r* = 0.588, *p* = 0.002). In slow-4 band, the fALFF value of the left precuneus was significantly positively correlated with MMSE score (*r* = 0.468, *p* = 0.018) and AFT score (*r* = 0.600, *p* = 0.002; Figure 3).

In the aMCI group, the fALFF value of the left orbital part of the inferior frontal gyrus was significantly positively correlated with AVLT long delay cued recall score (*r* = 0.506, *p* = 0.006; Figure 3).

## Discussion

In the stage of Obj-SCD, the neuropsychological decline includes two cognitive domains but is limited to one indicator of each domain. In the next stage of dementia progression, such as MCI, although cognitive impairment is aggravated, it may only be manifested in a single cognitive domain. Therefore, we speculate that the pathological characteristics of Obj-SCD may lead to a unique changing pattern, which is both similar and different from the next stage. Since the impairment in different cognitive domains will show different brain function activity changes, we made some efforts to select the cohort. For the Obj-SCD group, we chose individuals with impairments only in the memory domain and language domain, which is also the most common Obj-SCD individual. For MCI individuals, we chose a subgroup with only memory domain impairment, the aMCI group. The common feature of these two groups is the impairment of memory function. The difference lies in the degree of impairment, and the Obj-SCD group also has a slight impairment of language function. By comparing with HC and aMCI groups, we explored the functional activity changing pattern in Obj-SCD individuals. We performed correlation analysis between significant brain regions’ functional activities and neuropsychological test scores in order to verify this pattern.

### Similar Pattern Between the Obj-SCD and aMCI Groups

Compared with the HC group, we found similar functional activity enhancements in some local brain regions in the Obj-SCD and aMCI groups, including the left orbital part of the inferior frontal gyrus and the left median cingulate and paracingulate gyri.

The frontal lobe may be an essential area of cognitive maintenance associated with AD (Zeng et al., 2019). In terms of cognitive function, it may be involved in episodic memory and working memory (de Chastelaine et al., 2011; Matthews, 2015). Current evidence suggests that the orbitofrontal cortex is associated with memory impairment in AD and frontotemporal dementia (Liu et al., 2021). As an important node of working memory, the orbitofrontal gyrus may play an essential role in integrating and coordinating working memory maintenance, execution, and monitoring (Badre, 2008; Barbey et al., 2011; Costers et al., 2020). The cerebral accumulation of Aβ and the following neurovascular dysfunction were considered the most crucial pathogenesis of cognitive decline and dementia (Scheltens et al., 2016; Lim et al., 2018; Parodi-Rullán et al., 2020). Researchers (Hua et al., 2019) used arteriolar cerebral blood volume to explore the relationship between cerebral blood flow, APOE alleles, and Aβ accumulation in patients with MCI and found an increased blood volume in the orbitofrontal gyrus. Furthermore, they found the arteriolar cerebral blood volume in the orbitofrontal gyrus is closely related to local Aβ burden and APOE4; it also can predict cognitive decline within 2 years. Our study also found a similar increased local functional activity in the orbitofrontal gyrus in both Obj-SCD and aMCI groups, consistent with the increase of local blood flow in this area. This enhancement of functional activity may be a compensatory response to early cognitive decline. Considering the role of the orbitofrontal cortex in attention and impulsivity (Bari et al., 2020), it may also be a manifestation of nerve recruitment disorder.

The cingulate cortex is an essential structure in the human brain’s medial side, which plays a vital role in cognitive, motor, emotional, and other functional activities. According to the structure and function, the cingulate can be subdivided into several parts, in which the middle cingulate cortex is considered to be related to the function of the Frontoparietal Network (Vincent et al., 2008; Gilmore et al., 2015; Caruana et al., 2018). Specifically, the middle cingulate cortex is associated with emotion, behavior, motor, and somatosensory function; and has a close functional connection with prefrontal, premotor, and primary motor networks (Oane et al., 2020). Compared with the elderly with MCI, the elderly with normal cognition had a better connection between the middle cingulate and the superior frontal gyrus, frontal eye field, orbitofrontal cortex (Cera et al., 2019). Our study found increased functional activity on the middle cingulate cortex in Obj-SCD and aMCI groups, which may be intrinsically associated with the increased functional activity of the orbitofrontal cortex.

### Different Pattern Between the Obj-SCD and aMCI Groups

Some changes in local functional activities of the Obj-SCD group showed different patterns from the aMCI group. Compared with HC, the Obj-SCD group showed increased local functional activity in the right middle occipital gyrus, decreased local functional activity in the left precuneus and the left inferior temporal gyrus.

In the elderly with normal cognition, the connection between the occipital lobe and the posterior cingulate and precuneus is related to the tau protein, which may change in preclinical cognitive impairment (Quevenco et al., 2020). In patients with subjective cognitive decline, white matter damage across the frontal and occipital lobes was also found; this track is thought to be related to the gray matter damage such as the medial prefrontal cortex and posterior cingulate cortex in the early stage of AD (Luo et al., 2019). The close connection between the occipital lobe and the frontal lobe is consistent with finding abnormal functional activity in both the orbitofrontal cortex and the occipital cortex in our study. In people with normal cognition with Aβ deposition, it can be observed that the content of cerebrospinal fluid Progranulin is related to the thickening of the occipital cortex and cognitive decline; this result reflects that the neuroinflammatory reaction in the preclinical stage of Alzheimer’s disease may cause the compensatory performance in the occipital lobe (Batzu et al., 2020). Similarly, in individuals with subjective cognitive decline, an increase in the volume of occipital gray matter related to Aβ protein load was also found, and this structural change was associated with the aggravation of cognitive function symptoms (Valech et al., 2019).

Recent studies have shown that the temporal lobe is one of the critical areas of pathological changes in multiple types of dementia (Mak et al., 2020; Sanchez et al., 2021). In preclinical AD, tau protein deposition and Aβ burden in the inferior temporal gyrus is related to cognitive performance, mainly memory function (Schultz et al., 2018; Norton et al., 2020; Scott et al., 2020; Vila-Castelar et al., 2020). Tau accumulation in this area may be associated with hippocampal hyperactivity, which is not associated with Aβ (Huijbers et al., 2019). It is worth noting that in the normal elderly without Aβ deposition and in the elderly with cognitive impairment caused by small vessel disease, the lower functional connection of the inferior temporal gyrus is also related to the accumulation of tau protein (Franzmeier et al., 2019; Rabin et al., 2019). In addition, in normal older adults, the accumulation of tau protein in the inferior temporal gyrus is also associated with progressive thinning of the cortex in many brain regions, especially in the temporal lobe and parietal lobe (LaPoint et al., 2017). Therefore, the inferior temporal gyrus might be a susceptible region of detection of cognitive impaired risks.

In these studies, the changes in brain structure and functional activities are similar to our results of local functional changes observed in our research and may be related to pathological markers. However, considering that no relevant pathological marker detection was performed in our study, our results should still be interpreted with caution and cannot be directly explained by pathological changes.

### The Association Between Changing Pattern With Cognitive Function in Obj-SCD Group

In this study, the Obj-SCD group showed a unique changing pattern compared to the aMCI group. We analyzed the correlation between the functional activity of the significant brain regions found in the pattern and neuropsychological tests’ performance and found intimate associations.

The fALFF value of the right middle occipital gyrus was increased in the Obj-SCD group and was significantly negatively correlated with MMSE score and AFT score. These results suggested that the increase of local functional activity in this area may be related to the progression of the disease and may be a compensatory manifestation of general cognitive and language dysfunction. The functional activity of the left inferior temporal gyrus was positively correlated with the MMSE score, suggesting that the functional dysfunction was related to general cognitive function. The functional activity of the left precuneus was positively correlated with MMSE and AFT scores, suggesting that its dysfunction was related to the decline of general cognitive function and language function. The correlation between these brain regions and specific cognitive functions is consistent with previous studies. The abnormal brain areas of the Obj-SCD group are distributed in a relatively wide area, and there are differences between their correlations with different cognitive impairments. We speculate that it might because the internal pathological basis of Obj-SCD individuals is complicated. We also speculate that if the progress of different damage areas is inconsistent, Obj-SCD individuals’ outcomes may differ, but this speculation needs further follow-up studies to confirm.

Additionally, although the functional activity of the left orbital part of the inferior frontal gyrus changed in both the Obj-SCD and aMCI groups, only in the aMCI group the functional activity was positively correlated with AVLT long delay cued recall score. This can be attributed to the fact that the memory-binding impairment in the aMCI group is more severe than that in the Obj-SCD group.

## Conclusion

Obj-SCD individuals have a widely distributed pattern of local functional activity changes, and this changing pattern has some similarities with aMCI to a certain extent. However, there were also some differences. In this pattern, the functional changes of different brain regions have a close but different correlation with cognitive impairment, suggesting that there may be a complex pathological basis inside. This suggests that Obj-SCD may be a separate stage of cognitive decline before aMCI and is helpful to the study of preclinical cognitive decline.

## Limitations

The study still had some limitations. First, the sample size is not enough, resulting in insufficient sensitivity of the results. Second, there is no biomarker examination to explore the pathological mechanism. Therefore, the results should be interpreted with caution.

## Data Availability Statement

The raw data supporting the conclusions of this article will be made available by the authors, without undue reservation.

## Ethics Statement

The studies involving human participants were reviewed and approved by The Ethics Committee of Shanghai Jiao Tong University Affiliated Sixth People’s Hospital. The patients/participants provided their written informed consent to participate in this study.

## Author Contributions

LC: methodology, data analysis, visualization, and writing—review and editing. ZZ: methodology, data analysis, investigation, data curation, and writing—original draft. C-YZL: resources, project administration, review, and commentary. QG: resources, supervision, project administration, funding acquisition, review, and revision. All authors contributed to the article and approved the submitted version.

## Conflict of Interest

The authors declare that the research was conducted in the absence of any commercial or financial relationships that could be construed as a potential conflict of interest. The reviewer QZ declared a shared affiliation, with no collaboration, with one of the authors LC to the handling editor at the time of the review.

## Footnotes

^1^http://www.fil.ion.ucl.ac.uk/spm/software/spm12/

^2^http://restfmri.net/forum/restplus

## References

[B1] Badre D. (2008). Cognitive control, hierarchy, and the rostro-caudal organization of the frontal lobes. Trends Cogn. Sci. 12, 193–200. 10.1016/j.tics.2008.02.004 18403252

[B2] Barbey A. K. Koenigs M. Grafman J. (2011). Orbitofrontal contributions to human working memory. Cereb. Cortex 21, 789–795. 10.1093/cercor/bhq153 20724371 PMC3059885

[B3] Bari A. Xu S. Pignatelli M. Takeuchi D. Feng J. Li Y.. (2020). Differential attentional control mechanisms by two distinct noradrenergic coeruleo-frontal cortical pathways. Proc. Natl. Acad. Sci. U S A 117, 29080–29089. 10.1073/pnas.2015635117 33139568 PMC7682591

[B4] Batzu L. Westman E. Pereira J. B. Alzheimer’s Disease Neuroimaging Initiative (2020). Cerebrospinal fluid progranulin is associated with increased cortical thickness in early stages of Alzheimer’s disease. Neurobiol. Aging 88, 61–70. 10.1016/j.neurobiolaging.2019.12.012 31980280

[B5] Berron D. van Westen D. Ossenkoppele R. Strandberg O. Hansson O. (2020). Medial temporal lobe connectivity and its associations with cognition in early Alzheimer’s disease. Brain 143, 1233–1248. 10.1093/brain/awaa068 32252068 PMC7174043

[B6] Bi X.-A. Hu X. Wu H. Wang Y. (2020a). Multimodal data analysis of Alzheimer’s disease based on clustering evolutionary random forest. IEEE J. Biomed. Health Inform. 24, 2973–2983. 10.1109/JBHI.2020.2973324 32071013

[B7] Bi X.-A. Liu Y. Xie Y. Hu X. Jiang Q. (2020b). Morbigenous brain region and gene detection with a genetically evolved random neural network cluster approach in late mild cognitive impairment. Bioinformatics 36, 2561–2568. 10.1093/bioinformatics/btz967 31971559 PMC7178433

[B8] Bondi M. W. Edmonds E. C. Jak A. J. Clark L. R. Delano-Wood L. McDonald C. R.. (2014). Neuropsychological criteria for mild cognitive impairment improves diagnostic precision, biomarker associations, and progression rates. J. Alzheimers Dis. 42, 275–289. 10.3233/JAD-140276 24844687 PMC4133291

[B9] Caminiti S. P. Ballarini T. Sala A. Cerami C. Presotto L. Santangelo R.. (2018). FDG-PET and CSF biomarker accuracy in prediction of conversion to different dementias in a large multicentre MCI cohort. Neuroimage Clin. 18, 167–177. 10.1016/j.nicl.2018.01.019 29387532 PMC5790816

[B10] Caruana F. Gerbella M. Avanzini P. Gozzo F. Pelliccia V. Mai R.. (2018). Motor and emotional behaviours elicited by electrical stimulation of the human cingulate cortex. Brain 141, 3035–3051. 10.1093/brain/awy219 30107501

[B11] Cera N. Esposito R. Cieri F. Tartaro A. (2019). Altered cingulate cortex functional connectivity in normal aging and mild cognitive impairment. Front. Neurosci. 13:857. 10.3389/fnins.2019.00857 31572106 PMC6753224

[B12] Cerami C. Dodich A. Iannaccone S. Magnani G. Santangelo R. Presotto L.. (2018). A biomarker study in long-lasting amnestic mild cognitive impairment. Alzheimers Res. Ther. 10:42. 10.1186/s13195-018-0369-8 29695292 PMC5918759

[B13] Chen K. Huang L. Lin B. Zhou Y. Zhao Q. Guo Q. (2019). The number of items on each stroop test card is unrelated to its sensitivity. Neuropsychobiology 77, 38–44. 10.1159/000493553 30336464

[B14] Costers L. Van Schependom J. Laton J. Baijot J. Sjogard M. Wens V.. (2020). Spatiotemporal and spectral dynamics of multi-item working memory as revealed by the n-back task using MEG. Hum. Brain Mapp. 41, 2431–2446. 10.1002/hbm.24955 32180307 PMC7267970

[B15] Curiel Cid R. E. Crocco E. A. Duara R. Garcia J. M. Rosselli M. DeKosky S. T.. (2020). A novel method of evaluating semantic intrusion errors to distinguish between amyloid positive and negative groups on the Alzheimer’s disease continuum. J. Psychiatr. Res. 124, 131–136. 10.1016/j.jpsychires.2020.02.008 32146222 PMC10026350

[B16] de Chastelaine M. Wang T. H. Minton B. Muftuler L. T. Rugg M. D. (2011). The effects of age, memory performance, and callosal integrity on the neural correlates of successful associative encoding. Cereb. Cortex 21, 2166–2176. 10.1093/cercor/bhq294 21282317 PMC3155606

[B17] Dubois B. Hampel H. Feldman H. H. Scheltens P. Aisen P. Andrieu S.. (2016). Preclinical Alzheimer’s disease: definition, natural history, and diagnostic criteria. Alzheimers Dement. 12, 292–323. 10.1016/j.jalz.2016.02.002 27012484 PMC6417794

[B18] Folstein M. F. Folstein S. E. McHugh P. R. (1975). “Mini-mental state”. A practical method for grading the cognitive state of patients for the clinician. J. Psychiatr. Res. 12, 189–198. 10.1016/0022-3956(75)90026-6 1202204

[B19] Franzmeier N. Rubinski A. Neitzel J. Kim Y. Damm A. Na D. L.. (2019). Functional connectivity associated with tau levels in ageing, Alzheimer’s, and small vessel disease. Brain 142, 1093–1107. 10.1093/brain/awz026 30770704 PMC6439332

[B20] Gilmore A. W. Nelson S. M. McDermott K. B. (2015). A parietal memory network revealed by multiple MRI methods. Trends Cogn. Sci. 19, 534–543. 10.1016/j.tics.2015.07.004 26254740

[B21] Hua J. Lee S. Blair N. I. S. Wyss M. van Bergen J. M. G. Schreiner S. J.. (2019). Increased cerebral blood volume in small arterial vessels is a correlate of amyloid-beta-related cognitive decline. Neurobiol. Aging 76, 181–193. 10.1016/j.neurobiolaging.2019.01.001 30738323 PMC6438210

[B22] Huijbers W. Schultz A. P. Papp K. V. LaPoint M. R. Hanseeuw B. Chhatwal J. P.. (2019). Tau accumulation in clinically normal older adults is associated with hippocampal hyperactivity. J. Neurosci. 39, 548–556. 10.1523/JNEUROSCI.1397-18.2018 30482786 PMC6335746

[B23] Jack C. R. Jr. Bennett D. A. Blennow K. Carrillo M. C. Dunn B. Haeberlein S. B.. (2018). NIA-AA research framework: toward a biological definition of Alzheimer’s disease. Alzheimers Dement. 14, 535–562. 10.1016/j.jalz.2018.02.018 29653606 PMC5958625

[B44] Jia X.-Z. Sun J.-W. Ji G.-J. Liao W. Lv Y.-T. Wang J.. (2020). Percent amplitude of fluctuation: a simple measure for resting-state fMRI signal at single voxel level. PLoS One 15:e0227021. 10.1371/journal.pone.0227021 31914167 PMC6948733

[B24] Johansson B. Berg S. (1989). The robustness of the terminal decline phenomenon: longitudinal data from the Digit-Span Memory Test. J. Gerontol. 44, P184–P186. 10.1093/geronj/44.6.p184 2809113

[B25] Jones J. D. Uribe C. Bunch J. Thomas K. R. (2021). Beyond PD-MCI: objectively defined subtle cognitive decline predicts future cognitive and functional changes. J. Neurol. 268, 337–345. 10.1007/s00415-020-10163-4 32804281 PMC7855683

[B26] Katzman R. Zhang M. Y. Ouang Ya Q.-Y.-Q. Wang Z. Y. Liu W. T. Yu E.. (1988). A chinese version of the mini-mental state examination; impact of illiteracy in a Shanghai dementia survey. J. Clin. Epidemiol. 41, 971–978. 10.1016/0895-4356(88)90034-0 3193141

[B27] LaPoint M. R. Chhatwal J. P. Sepulcre J. Johnson K. A. Sperling R. A. Schultz A. P. (2017). The association between tau PET and retrospective cortical thinning in clinically normal elderly. NeuroImage 157, 612–622. 10.1016/j.neuroimage.2017.05.049 28545932 PMC5772972

[B28] Li Y. Yao H. Lin P. Zheng L. Li C. Zhou B.. (2017). Frequency-dependent altered functional connections of default mode network in Alzheimer’s disease. Front. Aging Neurosci. 9:259. 10.3389/fnagi.2017.00259 28824420 PMC5540901

[B29] Lim E.-Y. Yang D.-W. Cho A.-H. Shim Y. S. (2018). Cerebrovascular hemodynamics on transcranial doppler ultrasonography and cognitive decline in mild cognitive impairment. J. Alzheimers Dis. 65, 651–657. 10.3233/JAD-180026 30103317

[B30] Liu L. Roquet D. Ahmed R. M. Hodges J. R. Piguet O. Irish M. (2021). Examining prefrontal contributions to past- and future-oriented memory disturbances in daily life in dementia. Cortex 134, 307–319. 10.1016/j.cortex.2020.11.003 33333361

[B31] Luo C. Li M. Qin R. Chen H. Yang D. Huang L.. (2019). White matter microstructural damage as an early sign of subjective cognitive decline. Front. Aging Neurosci. 11:378. 10.3389/fnagi.2019.00378 32047428 PMC6997435

[B32] Mack W. J. Freed D. M. Williams B. W. Henderson V. W. (1992). Boston Naming Test: shortened versions for use in Alzheimer’s disease. J. Gerontol. 47, P154–158. 10.1093/geronj/47.3.p154 1573197

[B33] Mak E. Nicastro N. Malpetti M. Savulich G. Surendranathan A. Holland N.. (2020). Imaging tau burden in dementia with Lewy bodies using [^18^F]-AV1451 positron emission tomography. Neurobiol. Aging 101, 172–180. 10.1016/j.neurobiolaging.2020.11.006 33631469 PMC8209140

[B34] Manne S. Kondru N. Hepker M. Jin H. Anantharam V. Lewis M.. (2019). Ultrasensitive detection of aggregated alpha-synuclein in glial cells, human cerebrospinal fluid, and brain tissue using the RT-QuIC assay: new high-throughput neuroimmune biomarker assay for parkinsonian disorders. J. Neuroimmune Pharmacol. 14, 423–435. 10.1007/s11481-019-09835-4 30706414 PMC6669119

[B35] Matthews B. R. (2015). Memory dysfunction. Continuum 21, 613–626. 10.1212/01.CON.0000466656.59413.29 26039844 PMC4455839

[B36] Moguilner S. García A. M. Perl Y. S. Tagliazucchi E. Piguet O. Kumfor F.. (2020). Dynamic brain fluctuations outperform connectivity measures and mirror pathophysiological profiles across dementia subtypes: a multicenter study. NeuroImage 225:117522. 10.1016/j.neuroimage.2020.117522 33144220 PMC7832160

[B37] Morris J. C. (1993). The Clinical Dementia Rating (CDR): current version and scoring rules. Neurology 43, 2412–2414. 10.1212/wnl.43.11.2412-a 8232972

[B38] Norton D. J. Parra M. A. Sperling R. A. Baena A. Guzman-Velez E. Jin D. S.. (2020). Visual short-term memory relates to tau and amyloid burdens in preclinical autosomal dominant Alzheimer’s disease. Alzheimers Res. Ther. 12:99. 10.1186/s13195-020-00660-z 32825838 PMC7442980

[B39] Oane I. Barborica A. Chetan F. Donos C. Maliia M. D. Arbune A. A.. (2020). Cingulate cortex function and multi-modal connectivity mapped using intracranial stimulation. NeuroImage 220:117059. 10.1016/j.neuroimage.2020.117059 32562780

[B40] Pan P. Zhu L. Yu T. Shi H. Zhang B. Qin R.. (2017). Aberrant spontaneous low-frequency brain activity in amnestic mild cognitive impairment: a meta-analysis of resting-state fMRI studies. Ageing Res. Rev. 35, 12–21. 10.1016/j.arr.2016.12.001 28017880

[B41] Parodi-Rullán R. Ghiso J. Cabrera E. Rostagno A. Fossati S. (2020). Alzheimer’s amyloid beta heterogeneous species differentially affect brain endothelial cell viability, blood-brain barrier integrity, and angiogenesis. Aging Cell 19:e13258. 10.1111/acel.13258 33155752 PMC7681048

[B42] Petersen R. C. (2011). Clinical practice. Mild cognitive impairment. N. Engl. J. Med. 364, 2227–2234. 10.1056/NEJMcp0910237 21651394

[B43] Petersen R. C. Doody R. Kurz A. Mohs R. C. Morris J. C. Rabins P. V.. (2001). Current concepts in mild cognitive impairment. Arch. Neurol. 58, 1985–1992. 10.1001/archneur.58.12.1985 11735772

[B45] Pliskin J. I. DeDios Stern S. Resch Z. J. Saladino K. F. Ovsiew G. P. Carter D. A.. (2020). Comparing the psychometric properties of eight embedded performance validity tests in the rey auditory verbal learning test, wechsler memory scale logical memory, and brief visuospatial memory test-revised recognition trials for detecting invalid neuropsychological test performance. Assessment [Epub ahead of print]. . 10.1177/1073191120929093 32484371

[B46] Qualls C. E. Bliwise N. G. Stringer A. Y. (2000). Short forms of the Benton Judgment of Line Orientation Test: development and psychometric properties. Arch. Clin. Neuropsychol. 15, 159–163. 10.1093/arclin/15.2.159 14590559

[B47] Quevenco F. C. van Bergen J. M. Treyer V. Studer S. T. Kagerer S. M. Meyer R.. (2020). Functional brain network connectivity patterns associated with normal cognition at old-age, local beta-amyloid, tau, and APOE4. Front. Aging Neurosci. 12:46. 10.3389/fnagi.2020.00046 32210782 PMC7075450

[B48] Rabin J. S. Yang H.-S. Schultz A. P. Hanseeuw B. J. Hedden T. Viswanathan A.. (2019). Vascular risk and beta-amyloid are synergistically associated with cortical tau. Ann. Neurol. 85, 272–279. 10.1002/ana.25399 30565287 PMC6351182

[B49] Sanchez J. S. Becker J. A. Jacobs H. I. L. Hanseeuw B. J. Jiang S. Schultz A. P.. (2021). The cortical origin and initial spread of medial temporal tauopathy in Alzheimer’s disease assessed with positron emission tomography. Sci. Transl. Med. 13:eabc0655. 10.1126/scitranslmed.abc0655 33472953 PMC7978042

[B50] Scheltens P. Blennow K. Breteler M. M. B. de Strooper B. Frisoni G. B. Salloway S.. (2016). Alzheimer’s disease. Lancet 388, 505–517. 10.1016/S0140-6736(15)01124-1 26921134

[B51] Schultz S. A. Gordon B. A. Mishra S. Su Y. Perrin R. J. Cairns N. J.. (2018). Widespread distribution of tauopathy in preclinical Alzheimer’s disease. Neurobiol. Aging 72, 177–185. 10.1016/j.neurobiolaging.2018.08.022 30292840 PMC6422832

[B52] Scott M. R. Hampton O. L. Buckley R. F. Chhatwal J. P. Hanseeuw B. J. Jacobs H. I.. (2020). Inferior temporal tau is associated with accelerated prospective cortical thinning in clinically normal older adults. NeuroImage 220:116991. 10.1016/j.neuroimage.2020.116991 32512123 PMC7572623

[B53] Thomas K. R. Bangen K. J. Weigand A. J. Edmonds E. C. Wong C. G. Cooper S.. (2020). Objective subtle cognitive difficulties predict future amyloid accumulation and neurodegeneration. Neurology 94, e397–e406. 10.1212/WNL.0000000000008838 31888974 PMC7079691

[B54] Thomas K. R. Edmonds E. C. Eppig J. Salmon D. P. Bondi M. W. Alzheimer’s Disease Neuroimaging Initiative. (2018). Using neuropsychological process scores to identify subtle cognitive decline and predict progression to mild cognitive impairment. J. Alzheimers Dis. 64, 195–204. 10.3233/JAD-180229 29865077 PMC7263028

[B55] Thomas K. R. Osuna J. R. Weigand A. J. Edmonds E. C. Clark A. L. Holmqvist S.. (2021). Regional hyperperfusion in older adults with objectively-defined subtle cognitive decline. J. Cereb. Blood Flow Metab. 41, 1001–1012. 10.1177/0271678X20935171 32615887 PMC8054731

[B56] Valech N. Sánchez-Benavides G. Tort-Merino A. Coll-Padrós N. Olives J. Leãn M.. (2019). Associations between the subjective cognitive decline-questionnaire’s scores, gray matter volume, and amyloid-beta levels. J. Alzheimers Dis. 72, 1287–1302. 10.3233/JAD-190624 31707366

[B57] Vila-Castelar C. Muñoz N. Papp K. V. Amariglio R. E. Baena A. Guzmán-Vález E.. (2020). The Latin American Spanish version of the Face-Name Associative Memory Exam is sensitive to cognitive and pathological changes in preclinical autosomal dominant Alzheimer’s disease. Alzheimers Res. Ther. 12:104. 10.1186/s13195-020-00671-w 32912283 PMC7488408

[B58] Vincent J. L. Kahn I. Snyder A. Z. Raichle M. E. Buckner R. L. (2008). Evidence for a frontoparietal control system revealed by intrinsic functional connectivity. J. Neurophysiol. 100, 3328–3342. 10.1152/jn.90355.2008 18799601 PMC2604839

[B59] Worboys M. (2013). The Hamilton Rating Scale for Depression: the making of a “gold standard” and the unmaking of a chronic illness, 1960–1980. Chronic Illn. 9, 202–219. 10.1177/1742395312467658 23172888 PMC3837544

[B60] Zeng Q. Luo X. Li K. Wang S. Zhang R. Hong H.. (2019). Distinct spontaneous brain activity patterns in different biologically-defined Alzheimer’s disease cognitive stage: a preliminary study. Front. Aging Neurosci. 11:350. 10.3389/fnagi.2019.00350 32009939 PMC6980867

[B61] Zhao Q. Guo Q. Hong Z. (2013a). Clustering and switching during a semantic verbal fluency test contribute to differential diagnosis of cognitive impairment. Neurosci. Bull. 29, 75–82. 10.1007/s12264-013-1301-7 23322003 PMC5561862

[B62] Zhao Q. Guo Q. Li F. Zhou Y. Wang B. Hong Z. (2013b). The shape trail test: application of a new variant of the trail making test. PLoS One 8:e57333. 10.1371/journal.pone.0057333 23437370 PMC3577727

[B63] Zhao Q. Guo Q. Liang X. Chen M. Zhou Y. Ding D.. (2015). Auditory verbal learning test is superior to rey-osterrieth complex figure memory for predicting mild cognitive impairment to Alzheimer’s disease. Curr. Alzheimer Res. 12, 520–526. 10.2174/1567205012666150530202729 26027810

[B64] Zou Q.-H. Zhu C.-Z. Yang Y. Zuo X.-N. Long X.-Y. Cao Q.-J.. (2008). An improved approach to detection of amplitude of low-frequency fluctuation (ALFF) for resting-state fMRI: fractional ALFF. J. Neurosci. Methods 172, 137–141. 10.1016/j.jneumeth.2008.04.012 18501969 PMC3902859

[B65] Zuo X.-N. Di Martino A. Kelly C. Shehzad Z. E. Gee D. G. Klein D. F.. (2010). The oscillating brain: complex and reliable. NeuroImage 49, 1432–1445. 10.1016/j.neuroimage.2009.09.037 19782143 PMC2856476

